# 11p13 microduplication: a differential diagnosis of Silver–Russell syndrome?

**DOI:** 10.1186/s13039-024-00672-6

**Published:** 2024-03-14

**Authors:** Asmaa K. Amin, Jeremias Krause, Thomas Eggermann

**Affiliations:** 1 Department of Human Genetics, Medical Research Institute, Alexandria University, Alexandria, Egypt; 2 Institute for Human Genetics and Genome Medicine, Medical Faculty, RWTH Aachen University, Pauwelsstr. 30, 52074 Aachen, Germany

**Keywords:** 11p13 microduplication syndrome, Silver–Russell syndrome, *SLC1A2*

## Abstract

**Background:**

Silver–Russel syndrome (SRS) is a congenital disorder which is mainly characterized by intrauterine and postnatal growth retardation, relative macrocephaly, and characteristic (facial) dysmorphisms. The majority of patients shows a hypomethylation of the imprinting center region 1 (IC1) in 11p15 and maternal uniparental disomy of chromosome 7 (upd(7)mat), but in addition a broad spectrum of copy number variations (CNVs) and monogenetic variants (SNVs) has been reported in this cohort. These heterogeneous findings reflect the clinical overlap of SRS with other congenital disorders, but some of the CNVs are recurrent and have therefore been suggested as SRS-associated loci. However, this molecular heterogeneity makes the decision on the diagnostic workup of patients with SRS features challenging.

**Case presentation:**

A girl with clinical features of SRS but negatively tested for the IC1 hypomethylation and upd(7)mat was analyzed by whole genome sequencing in order to address both CNVs and SNVs in the same run. We identified a 11p13 microduplication affecting a region overlapping with a variant reported in a previously published patient with clinical features of Silver–Russel syndrome.

**Conclusions:**

The identification of a 11p13 microduplication in a patient with SRS features confirms the considerable contribution of CNVs to SRS-related phenotypes, and it strengthens the evidence for a 11p13 microduplication syndrome as a differential diagnosis SRS. Furthermore, we could confirm that WGS is a valuable diagnostic tool in patients with SRS and related disorders, as it allows CNVs and SNV detection in the same run, thereby avoiding a time-consuming diagnostic testing process.

## Background

Duplications in 11p13 have been documented for several times in the literature (for review: [1]) and in databases (DECIPHER, https://www.deciphergenomics.org/), the majority of them are associated with neurodevelopmental disorders. However, common breakpoints have not yet been identified and the sizes of the duplications differ, accordingly the phenotypes of the carriers are heterogenous. In contrast, a deletion syndrome in 11p13 has already been suggested (distal 11p13 deletion syndrome, OMIM #616902, genomic coordinates (GRCh38): 11:31,000,001-36,400,000) [2]. The syndrome shows incomplete penetrance, and ranges from general developmental delay, speech and language disorders to autism spectrum disorders. Despite the previously published 11p13 microduplication cases, recurrent breakpoints in 11p13 have not yet been identified, and the patients´ phenotypes are influenced by the variable loss of additional genes.

Among the 11p13 microduplication carriers reported so far, one patient with Silver-Russell syndrome (SRS) features has been described [1] (Table 1). He exhibited intrauterine and postnatal growth retardation, macrocephaly and facial aspects which are clinical key features for this congenital disorder [3]. SRS is primarily an imprinting disorder associated with molecular defects affecting genomically imprinted regions on chromosomes 11p15, 7 and 14q32. Due to its clinical heterogeneity and the non-specificity of its key features there is a broad overlap with further inborn disorders. Accordingly, a significant number of patients with features of SRS carry clinically relevant copy number variants (CNVs) (for review: [4]), and approaches targeting CNVs should therefore be included in the molecular diagnostic workup [3].

**Table 1:** Clinical findings in the two 11p13 duplication patients with SRS features

Patient	SRS key features*	Palumbo et al. [1]	Present case
Size		4.3 Mb	2.76 Mb
Growth	SGA*	Yes	Yes
	Postnatal growth failure*	Yes	Yes
	Feeding difficulties and/or lower BMI*	No	No
Facial gestalt	Relative macrocephaly*	Yes	No
	Protruding forehead*	No	Yes
	Triangular face	Yes	Yes
	Retrognathia	Yes	No
	Downturned corners of the mouth	Yes	No
Body/limbs	Body asymmetry*	Yes	Slight
	Muscular hypotonia	Yes	No
	Clinodactyly V	Yes	Yes
	Scapular winging	Yes	Yes
Development	Psychomotoric delay	Yes	Yes
	Speech delay	Yes	Yes
NHS score		4	3 (4)

*Parameters covered by the clinical SRS score [3]; the explanation to list the total scoring of 4 for the Netchine–Harbison Score (NHS) in parenthesis is provided in the text; *SGA* small for gestational age; *BMI* body mass index

**Fig. 1:**
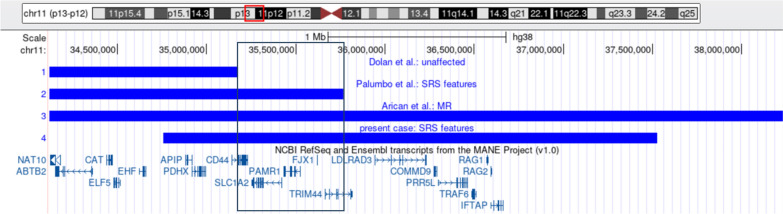
UCSC custom track illustrating the overlap of the duplications in 11p13 in patients with SRS features (hg38). Only duplications from those patients with copy number variants spanning the smallest region of interest are shown [1, 5, 9]. The smallest common region of overlap comprises approximately 1 Mb and harbors five genes

In a patient referred for SRS testing we now identified a 2.76 Mb duplication which overlaps with the duplicated regions in the patient with SRS features published by Palumbo et al. and other cases from the literature and databases (for review: [1, 5] (Fig. 1). By comparing the breakpoints of these patients, we narrowed down the candidate region for a putative 11p13 microduplication syndrome.

## Case presentation

The girl was the third child of healthy nonconsanguineous Egyptian parents. Family history is empty. Parental height was in the normal range (mother: 172 cm, father: 174 cm). 

The patient was born spontaneously at term after an uneventful pregnancy with a birth weight of 1750 g (z-3.81). Neither feeding difficulties nor hypotonia were reported. At the age of 4 5/12 years, length was 96 cm (z − 2.29), weight 11 kg (z − 3.76) and occipital frontal circumference 46.6 cm (z − 3.11). 

**Fig. 2:**
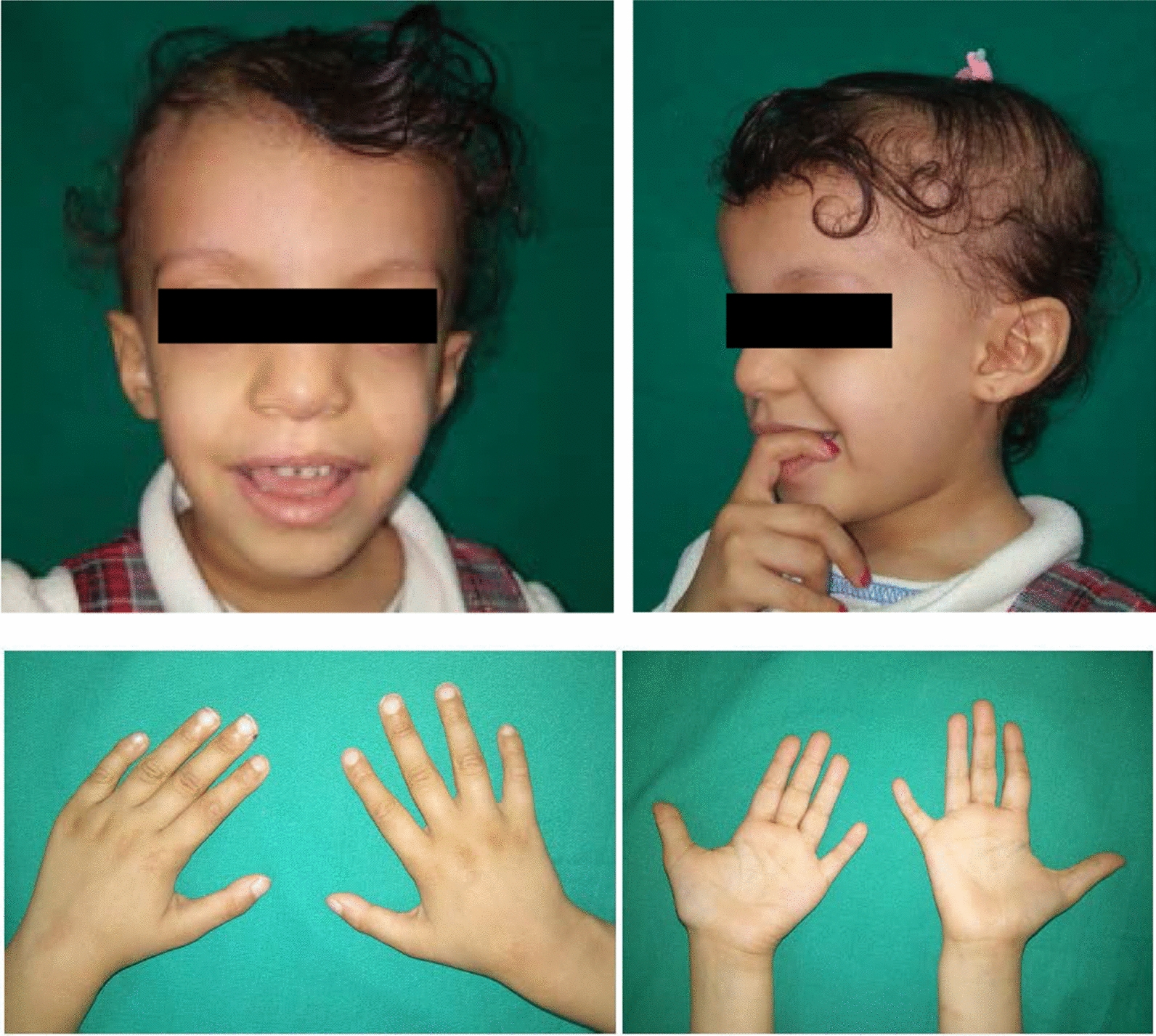
The patient with 11p13 duplication at the age of 4.5 years

Facial features at that age (Fig. 2, Table 1) included a prominent forehead, a triangular face, downslanting palpebral fissure, hypertelorism, a broad prominent nasal root and bridge, and posteriorly inclined ears with small ear lobule. Mild clinodactyly V and brachydactyly V were documented, as well as broad big toes and clinodactyly of 3rd and 4th toes. Multiple café-au-lait spots and hypopigmented areas were observed.

Endocrinological testing revealed normal basal growth hormone values (3.9 ng/ml; normal: up to 7), but a weak response after stimulation with clonidine. X ray of the left hand at the age of 21 months showed a retarded bone age of 12 months. Developmental milestones were reported as delayed by not further documented. 

In summary, the patient exhibited features of SRS, and the clinical Netchine-Harbison score revealed at least three out of six criteria (intrauterine and postnatal growth retardation, prominent forehead, no relative macrocephaly, feeding difficulties; the patient also displayed a slight body asymmetry (Table 1: the NHS scoring of 4 is given in parentheses).

The patient was negatively tested for the typical molecular alterations observed in SRS (Loss of methylation in imprinting center 1 (IC1 LOM), maternal uniparental disomy of chromosome 7 upd(7)mat, 14q32 alterations). Chromosomal analysis revealed a normal karyotype (46,XX).

## Material and methods

Whole genome enrichment of the DNA samples of the patients and her parents was conducted by using a DNA PCR-free assay (Illumina Inc. San Diego, CA, USA) and sequencing was performed on a NovaSeq 6000 (S4 Reagent Kit v1.5) (Illumina Inc.). Data analysis was performed with the Illumina DRAGEN-Pipeline (Version: 07.021.645.4.0.3) in comparison with the human reference genome (hg38). Variant filtering annotation was performed using KGGSeq (v1.0,20/jun/2018) discarding variants with a minor allele frequency higher than 0.75% in public databases (i.e. gnomAD, 1000 Genome Project, Exome Variant Server). Variant priorisation and evaluation of pathogenicity was based on different prediction tools (CADD, Polyphen, SIFT, MutationTaster) and variant frequency in public databases. The WGS data were analysed in respect to single nucleotide variants (SNVs), CNVs, uniparental disomies and (known pathogenic) repeat expansions. Pathogenicity of SNVs and CNVs was based on standardized guidelines of the American College of Medical Genetics (ACMG) [6, 7].

The duplication was confirmed by molecular karyotyping using a SNP array (CytoScan™ HD Array, Life Technologies, Carlsbad/USA).

## Discussion and conclusion

In a patient with SRS features but negatively tested for the characteristic molecular SRS disturbances, a de-novo 2.76 Mb duplication was identified by trio whole genome sequencing (trio-WGS), seq[hg38]11p13(34,759,059-37,524,365)dup, dn. The trio-WGS data did not provide evidence for any other clinically relevant SNVs, small CNVs (< 50 kb), uniparental disomy or pathological repeat expansions. 

Based on the available data and knowledge, pathogenicity evaluation of the CNV according to the recently published ACMG recommendation [7] classified the alteration as variant of unknown significance. However, due to the de-novo occurrence of the CNV, the documentation of another patient with an overlapping duplication and a similar phenotype [1] , and the absence of duplications of the region among healthy controls (DGV, http://dgv.tcag.ca/dgv/app/home) we suggest the variant as likely disease-causing. This classification is additionally corroborated by the observation that patients with SRS like phenotypes exhibit a broad range of CNVs, but that several of them reoccur and affect the same regions, e.g. 1q21, 15q26, 17p13.3, and 22q11 [4, 8]. However, these heterogeneous genetic findings in patients with SRS features confirms the unspecifity of its clinical signs, and the appropriateness of a comprehensive diagnostic workup. Clinically, the presence of relative macrocephaly and protruding forehead has been suggested to distinguish clinical SRS from other growth retardation syndromes after exclusion of the typical molecular SRS findings [3]. In fact, our patient and the comparable case from the literature [1] only exhibit one of these features each and therefore do not fully suit the definition of clinical SRS. However, this also counts for molecularly confirmed SRS patients, thereby illustrating the difficulty of clinical diagnosis of SRS.

By search for 11p13 duplications in the literature [1, 5] and databases (DECIPHER) several cases with different neurodevelopmental and other features could be identified. However, a detailed comparison with the size and gene content in our patient showed that the same variant has not yet been published (Fig. 1). Therefore, recurrent breakpoints are not obvious, and the clinical range of 11p13 duplications can at least in part be explained by the different sizes and gene contents of the variants.

The 2.76 Mb duplication in our patient represents the smallest duplication in the region, and by considering the previously published patient with SRS features [1] and a duplication carrier without clinical features [9] it allows to narrow down the smallest common region of overlap in the two patients with SRS features to 1 Mb. This region harbors five protein-coding genes, four of them have not yet been identified as disease-causing (*PAMR1, FJX1*) or are associated with clinical features not fitting with that in the two deletion carriers (*CD44, TRIM44*). Pathogenic loss-of-function variants of the fifth gene, *SLC1A2,* are associated with a developmental and epileptic encephalopathy (DEE41, OMIM # 617105) and poor overall growth, a gain-of-function variant in this gene has recently been suggested to be associated with epileptic encephalopathies [10]. In fact, our patient did not exhibit clinical features consistent with the latter condition, but a complementing contribution of all the affected genes to the phenotype is well conceivable.

In summary, we suggest duplications of a 1 Mb region within 11p1 as a new microduplication syndrome with a clinical overlap with SRS. Furthermore, we could confirm that trio-WGS is a valuable diagnostic tool which allows a one-step analysis to identify CNVs and SNVs in patients with clinically heterogeneous features like SRS and related disorders, thereby avoiding a time-consuming diagnostic testing process.

## Data Availability

Data are available on request.
